# A pseudo-ketogenic sugar-ghee-enriched diet induces metabolic and immune alterations in rats: a model of flawed ketogenic diet practice

**DOI:** 10.3389/fvets.2025.1582086

**Published:** 2025-10-06

**Authors:** Farah Ismail, Mohammad Khalifeh, Wael Hananeh, Batool Khataybeh, Rasha Al-Azaizeh, Muath Q. Al-Ghadi

**Affiliations:** ^1^Department of Basic Medical Veterinary Sciences, College of Veterinary Medicine, Jordan University of Science and Technology, Irbid, Jordan; ^2^Department of Molecular Biology and Genetic Engineering, College of Science, Jordan University of Science and Technology, Irbid, Jordan; ^3^Department of Veterinary Pathology and Public Health, Jordan University of Science and Technology, Irbid, Jordan; ^4^Department of Nutrition and Food Technology, Jordan University of Science and Technology, Irbid, Jordan; ^5^Department of Zoology, College of Science, King Saud University, Riyadh, Saudi Arabia

**Keywords:** ketogenic diet, Wistar rats, ghee, lipid profile, histopathologically, immune response

## Abstract

**Introduction:**

The ketogenic diet (KD) has gained popularity due to its reported benefits on weight loss and metabolic health. However, in real-world settings, KD is frequently misapplied individuals often continue consuming sugar or fail to calculate macronutrient ratios accurately. These flawed patterns may still result in weight loss but carry unclear long-term effects on metabolism and immune function.

**Methods:**

The present study aimed to simulate one such misapplication by developing a sugar-ghee-enriched diet (SGED) for Wistar male rats, rich in animal-derived fat and added sugar. The diet provided approximately 31.7% of energy from fat with a ketogenic ratio of 0.21:1, well below the threshold for nutritional ketosis. Rats were divided into control and SGED groups and fed their respective diets for 33 days. We examined parameters including body weight, visceral fat deposition, serum lipid levels, selected cytokines (IL-6, TNF-*α*, IL-10, TGF-*β*), and performed histological examinations of the liver, kidney, and intestinal tissues.

**Results:**

SGED-fed rats showed a reduction in total body weight but exhibited a significant increase in visceral fat deposition and a dyslipidemic profile, marked by elevated serum triglyceride, cholesterol, vLDL levels, and atherogenic index. Immune modulation was also observed, with increased levels of TNF-*α*, IL-10, and TGF-*β*, and a decrease in IL-6. No major histopathological changes were found in the examined organs.

**Discussion:**

To our knowledge, this is the first study to introduce an experimental rat model representing pseudo-ketogenic dietary (PKD) behavior, characterized by high animal fat intake combined with added sugar, leading to superficial weight loss without achieving ketogenic thresholds. The SGED model reveals potential risks for adverse immune and metabolic outcomes, emphasizing the need to address flawed interpretations of ketogenic dieting.

## Graphical abstract

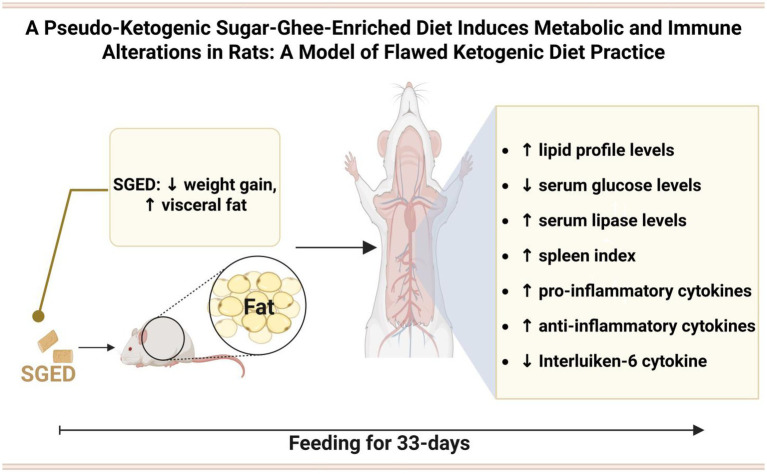

## 1 Introduction

Obesity and metabolic disorders are achieved in rat models by excessive consumption of diets rich in both fat and sugar (1). Traditionally, high-fat/high-sugar (HFHS) animal models have been employed in understanding diet-induced obesity, while a parallel trend in human behavior has recently emerged with the widespread popularity of the ketogenic diet (KD) (2). KD describe a type of diet that is composed of a low carbohydrate, high fat dietary approach originally developed for epilepsy management, then adopted for weight loss and metabolic control. The classical ketogenic diets require to adhere strictly to macronutrient ratios that typically providing 90% of energy from fat, 8–10% from protein, and less than 5% from carbohydrates, which overall results in a ketogenic ratio (fat to protein + carbohydrate) to be of ≥3:1 (3).

This KD has been extensively used for weight loss and metabolic control. Several forms of this type of diet also been developed, each with varying macronutrient ratios, including the standard ketogenic diet (SKD), cyclical ketogenic diet (CKD), targeted ketogenic diet (TKD), and high protein ketogenic diet (HPKD), each with varying macronutrient ratios. Typically, KD formulations range from 4:1 to 1:1 in fat to carbohydrate plus protein ratio (3). In contrast, the term ‘high-fat diet’ (HFD) in rodent studies generally refers to diets where 35–70% of calories come from fats, often leading to obesity and insulin resistance (4, 5). In a ketogenic diet, individuals must consume 60–75% of fat, carbohydrates not exceeding 5%, and the rest should be proteins from different sources (6). However, proper adherence to this diet is notoriously difficult. In practice, many individuals follow what may be described as a pseudo-ketogenic pattern, consuming high levels of fat (often from animal sources) while continuing to eat sugar or failing to calculate macronutrient ratios accurately. This under-mines the metabolic shift toward ketosis and often results in a diet that resembles ketogenic eating in appearance but not in effect. In a survey conducted among ketogenic diet adopters, over 54% reported having weekly cheat meals, while 42.5% did not follow any structured plan. Surprisingly, weight loss was still reported by 96.9% of respondents, indicating that misapplication may produce superficial results while masking deeper metabolic imbalances (7). Importantly, discontinuing or misapplying the ketogenic diet has been associated with weight regain, worsened inflammatory profiles, and increased central adiposity, particularly visceral fat accumulation (8–10). This rebound pattern where weight is regained and redistributed toward abdominal fat is especially common in individuals who fail to maintain ketosis or reintroduce sugars during or after the diet. Such changes are also linked to elevations in pro-inflammatory cytokines, suggesting a potential risk of long-term metabolic dysregulation (11).

These findings highlight the need for experimental models that accurately simulate pseudo-ketogenic behavior in humans. To address this, we developed the Sugar-Ghee-Enriched Diet (SGED), which mirrors a commonly observed misapplication: the consumption of animal-derived fats (ghee) along with added sugar, without maintaining the macronutrient balance required for ketosis. The SGED provides only 31.7% of energy from fat and has a ketogenic ratio of 0.21:1, making it non-ketogenic despite its high-fat appearance. The SGED model was carefully designed to walk a fine line, avoiding overt weight gain and obesity, which would classify it as a typical high-fat diet (HFD) model, while still eliciting modest weight loss despite miscalculated macronutrient ratios, thereby capturing the immune metabolic consequences of flawed ketogenic diet application.

This study investigates the metabolic and immunological effects of SGED in rats, with a particular focus on visceral fat accumulation, lipid profile disruption, and immune modulation, as a model for understanding the risks associated with pseudo-ketogenic dietary practices.

## 2 Materials and methods

### 2.1 Animals and experimental design

Male Wistar rats (*n* = 96), aged 4–5 weeks, weighing (90–120 g), were obtained from the Animal House Unit, Jordan University of Science and Technology, Irbid, Jordan. Female rats were excluded due to potential hormonal influences on the measured parameters. Rats were housed in plastic cages (three rats per cage, each identified by color labels) with proper ventilation and under optimum hygienic conditions, in a light–dark cycle (lights on at 07:00 h) and at a temperature of 19–22°C and 30–40% humidity. The animals are provided water and a different rat diet ad libitum. Two separate experiments were conducted using a total of 96 rats. The first experiment involved 58 rats, and the second involved 38. Across both experiments, the rats were randomly assigned to one of two dietary groups. The first group, consisting of 60 rats (40 from the first experiment and 20 from the second), received a specially formulated enriched diet called SGED. The second group, comprised of 36 rats (18 from each experiment), received standard rat food.

The study design was approved by the Animal Care and Use Committee of Jordan University of Science and Technology, which adheres to the Institute of Laboratory Animal Resources (ILAR) guidelines (12).

### 2.2 Diet preparation

Control rats received a conventional diet containing (g%): 5% total fat, 62% carbohydrates, 20% proteins, 13% fiber, ash, and other ingredients. The formulated SGED was prepared by adding certain ingredients to the standard feed chow which its fat source is mainly of plant-origin. First, 150 g of ghee (Al-Aseel, UAE) was weighed and melted in the microwave for 1 min. Then, it was poured on 1,000 g of basal diet and mixed for 5 min. Then, 150 g of table sugar (Nader sugar, Jordan) and 30 g of table salt (Sasi, Jordan) were dissolved in 200 mL of tap water. The mixture (water, sugar, and salt) was heated in the microwave for 3–5 min until all the ingredients melted well. After the mixture has cooled, 5 mL of vitamin and mineral supplements (Kiddi Pharmanton, Thailand) were added and stirred well. The vitamins and minerals present in the product contained per 15 mL a complex of: calcium (43.35 mg), vitamin B1 (1 mg), vitamin B2 (1.15), vitamin B6 (2 mg), vitamin D3 (133.35 IU), vitamin E (5 mg), nicotinamide (vitamin PP) (6.65 mg), dexpanthenol (3.35 mg), and lysine hydrochloride (100 mg). The final mixture (water, sugar, salt, and vitamin) was added to the chow containing ghee and stirred well, then dried in the microwave for 5 min. Consequently, the SGED diet contains (g%): 15% total fat, 57.9% carbohydrates, 15% proteins, 2.3% salts, 9.8% fiber, ash, and other ingredients. The SGED diet was prepared every other day and was preserved in room temperature until served. This ghee is manufactured from 100% cow’s milk contains an average value per 100 g contain 700 kilojoules (Kj) with 100% total fat as saturated (70%), monounsaturated (25%), and polyunsaturated 5% as well as 330 mg cholesterol. The ghee used in this study was manufactured from 100% bovine cream using traditional heat clarification methods (without chemical processing or solvents). This traditional process yields ghee with a fat content exceeding 99%, while the remaining fraction consists of moisture and trace milk solids (SNF). The rats remained on this diet until the end of the experiments (33 days).

The caloric distribution of macronutrients was calculated using Atwater conversion factors: 9 kcal/g for fat, 4 kcal/g for protein, and 4 kcal/g for carbohydrates The ketogenic ratio (KR) for all diets was calculated based on the standard clinical formula: fat in grams divided by the sum of carbohydrate and protein in grams (fat/[protein + carbohydrate]) (13). For the reference diets, the macronutrient composition was extracted from the original articles, but the KR was not explicitly reported. Therefore, KR values were manually computed to enable consistent comparison across all diet types (13) (Table 1). The energy content and macronutrient contributions for each experimental diet were calculated and compared with energy content of several ketogenic diet models and HFD present in literature as follows in Table 1.

**Table 1:** Comparative macronutrient energy composition, energy density, and ketogenic ratios of the experimental diets and reference diets from published studies.

Diet type	% Kcal from fat	% Kcal from protein	% Kcal from carbohydrates	Energy density (kcal/g)	Ketogenic ratio (F(g)/[P(g) + C(g)])	Reference
Normal diet (ND)	11.8%	23.5%	64.7%	3.8	0.06: 1	Current study
Pseudo-ketogenic diet (SGED/PKD)	29.0%	12.9%	49.7%	4.65	0.21: 1	Current study
High-fat diet (HFD)	60.0%	15.3%	24.7%	~5.24	1.5: 1	Buettner et al (4)
Classical ketogenic diet (CKD)	90.0%	4.8%	5.2%	6.7	4: 1	Barzegar et al. (3)
Standard ketogenic diet (SKD)	75.0%	20.0%	5.0%	~6.0	3: 1	Shilpa et al. (62)

### 2.3 Animal weight and the feed conversion ratio (FCR)

The body weight and feed intake were measured daily using a digital weighing scale (PGA OER-LIKON AG, Zurich, Switzerland). Although weight gain was recorded daily, data were summarized and presented as averages over 3-day intervals.

The weight gain percentage (WG%) was calculated for each rat every 3 days using the following equation:


$$\mathrm{WG}\%=[(\begin{array}{l} \text{Average weight}\;\mathrm{at}\;3-\mathrm{day}\;\\ \text{interval}-\text{initial weight} \end{array})/\text{initial weight}]\times 100$$

The mean WG% for all rats in each group was then computed and compared between groups.

The feed conversion ratio (FCR) was calculated based on the total feed intake and total weight gain of the three animals housed per cage over every 3-day interval, as follows:


$$\begin{array}{l} \mathrm{FCR}=\text{Total feed intake}\;\mathrm{per}\;\text{cage}\;(g)/\text{total weight gain}\;\\ \;\text{of the three rats}\;\mathrm{per}\;\text{cage}\;(g) \end{array}$$

These values were used to assess feed efficiency and metabolic changes between the SGED and control groups.

### 2.4 Determination of the serum lipid profile, glucose, lipase, and liver enzymes concentration

At the end of the experiment and before the animals’ euthanization, rats were fasted for 12 h and then decapitated using guillotines. The blood was then collected in plain tubes. Then, the serum was separated by centrifugation at 10,000 g at 4°C for 15 min. The serum was kept at −20°C until the time of analysis. Lipid profile serum total cholesterol, low-density lipoprotein (LDL) cholesterol, high-density lipoprotein (HDL), triglycerides (TG), glucose, lipase, and liver enzyme alanine transaminase (ALT), and aspartate aminotransferase (AST) analysis were performed according to the manufacturer’s recommendation. AGAPPE kit was used; for HDL: Code number 52013001, whereas for LDL, cholesterol and TG: Code number: 11579, 11,805, 11,528, respectively, BioSystems, Spain. Serum glucose levels were measured using an enzymatic photometric test in line with the manufacturer’s recommendations (Code number: CH0280, ARCOMEX, Jordan). Using a spectrophotometer (UV/VIS spectrometer T80+, United States), the absorbance (A) and the standard were measured for all samples at 500 nm against a blank reagent. Except for HDL, where its reaction absorbance was measured at 505 nm. The formula included in the kits was used to determine the sample’s cholesterol concentration. Very-low-density lipoprotein cholesterol (VLDL) was estimated using the Friedewald equation. Moreover, lipase concentration was calculated using the Biochemical Enterprise kit (Code number: LIP3542, Biochemical Enterprise, Italy). Whereas, serum liver enzyme was analyzed using Spinreact kit (Code number: 1001172, 1,001,162, respectively, Spinreact kit, Spain).

### 2.5 Histological changes of the tissues and organ weight in response to SGED and tissue indices calculation

Representative tissue samples from the liver, spleen, kidneys, lungs, and visceral fat were cut and fixed in 10% formaldehyde for 24 h. The organs were weighed before being preserved in formalin. Visceral fat weights were calculated as the sum of the fats weight of the retroperitoneal, perineal, and perigonadal. The body fat as well as organ indices were calculated by dividing the weight of these tissues by the final body weight at day 33. Tissues were taken for microscopic analysis to show if this formulated fat based diet caused any abnormal changes. Later, the fixed tissues (10% formaldehyde) were dehydrated in a series of ethanol treatments, starting from the 70% storing solution, and then cleared in xylene. The blocks were serially sectioned at 5 μm with a rotary microtome (Motorized rotary microtome, United States). Then, the hematoxylin and eosin (H&E) staining technique was used to stain the sections and evaluate their general morphology.

To evaluate organ size relative to body weight, indices were calculated for selected tissues using the following formula:


Organ index(%)=(Organ weight(g)/final body weight(g))×100

This formula was applied to compute the visceral adiposity index, hepatosomatic index, splenic index, renal index (RSI), and pulmonary index.

The Atherogenic Index, a marker for cardiovascular risk. It reflects the balance between atherogenic and protective lipoproteins, with higher values indicating increased cardiovascular risk. This index was calculated using serum lipid profile data based on the following formula:


$$\mathrm{AI}=\log_{10}(\text{triglycerides}\;(\text{nmol}/L)/\mathrm{HDL}-\text{cholesterol}\;(\text{nmol}/L))$$

### 2.6 Determination of the serum cytokines concentration by ELISA

To determine the cytokine level in rats’ serum, a sandwich ELISA was performed according to the manufacturer’s recommendation (Sincere Biotech, China). The following cytokine concentrations were determined: IL-6, IL-10, TNF-*α*, and TGF-*β*, all of which were assayed in a similar procedure.

### 2.7 Statistical analysis

All values are displayed as mean ± S.E.M. Statistical analysis was accessed using OpenEpi.^1^ Significant differences among means were tested using the Student’s *t*-test. Differences were considered significant at *p*-value < 0.05.

## 3 Results

### 3.1 Body weight changes and feed conversion rate

**Figure 1:**
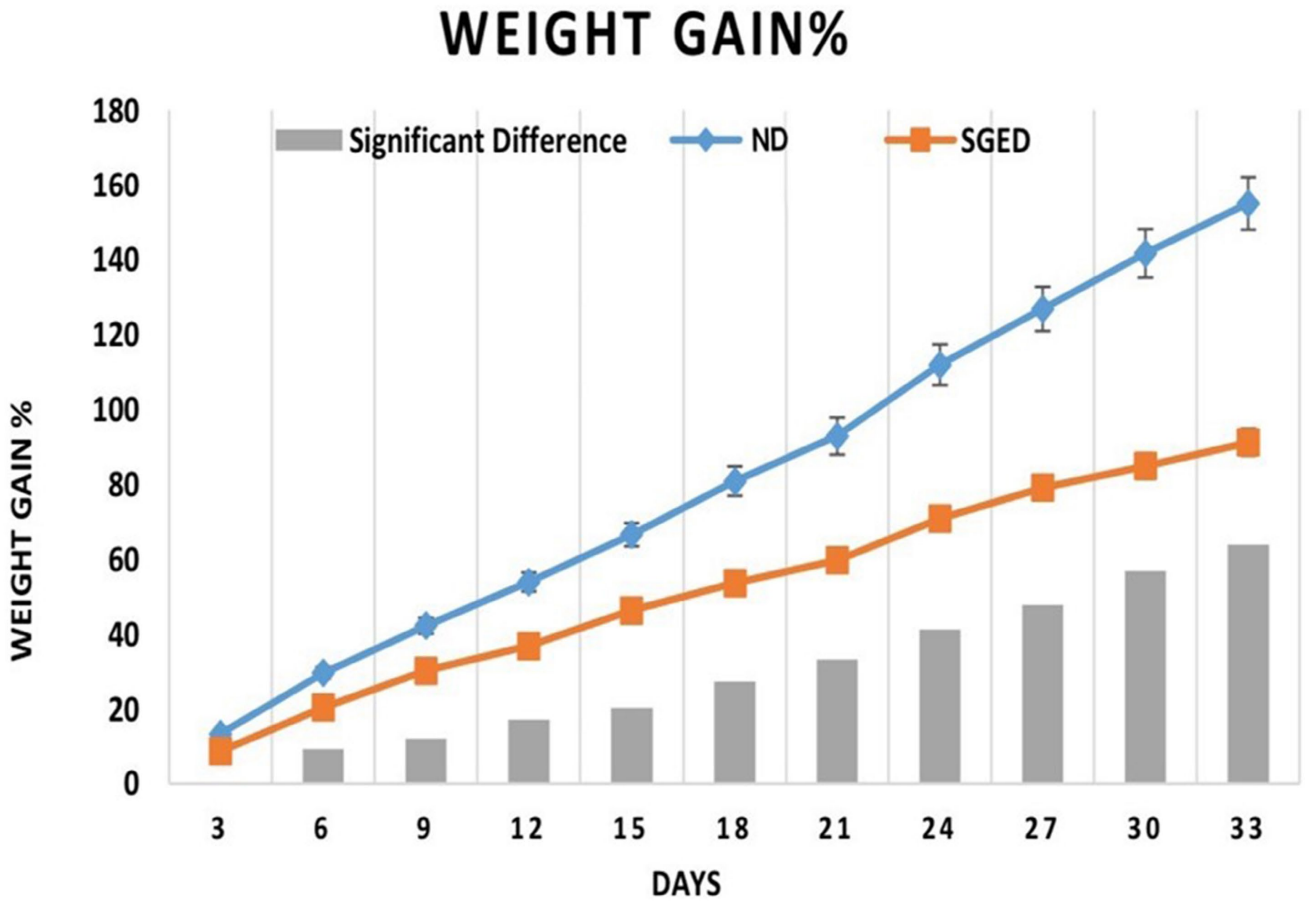
The 3-days weight gain percentage and the differences in weights in response to consume sugar-ghee enriched diet (SGED) (60 rats) and a normal diet (control) (36 rats). The results represent data from two separate experiments. Rats were weighed daily, but the results were presented as average weight gain every 3 days in reference to the initial weight. Each time point showed the mean of the weight gain percentage ± S.E. The significant difference (SGED-ND) with a *p*-value less than 0.05 is presented by the columns in the figure.

After a 33-day experimental observation of a group of rats fed a normal diet (control) and a sugar-ghee-enriched diet (SGED), results revealed that the weight gains of rats fed a normal diet (control) were significantly higher than those rats fed SGED. As shown in Figure 1, rats in both groups showed similar weight gain in the first 3 days. In the next 3 days until day 6 and onward till day 33, it became clear that the rats fed a normal diet significantly gained more weight compared to rats fed SGED. Therefore, the rats fed SGED gained about 91.1% more of their original weight at the end of the experiment, whereas the rats fed the normal diet gained almost 92.8% more of their original weight on day 21 of the experiment. At the end of the experiments, the weight gains of the control groups reached 155%. The feed consumption of the rats was approximately equal in both groups, with preference toward SGED being above the FCR reported in control rats, which was to be significant at days 12 and 33.

### 3.2 Changes of the amount of visceral fat and organs weight and pathological evaluations

The effect of consuming SGED for 33 days on visceral fat mass was investigated. The SGED rats group had a significantly higher amount of visceral fat compared to normal diet rats group. As shown in Table 2, the rats that consumed SGED showed nearly four times increases in body fat and adiposity index compared to the normal diet group. Although liver and lung weights were significantly lower in the SGED group compared to the normal diet-fed group (Table 2), their pulmonary and hepatosomatic indices were similar. Kidney and spleen weight were similar in both groups but the splenic index was slightly higher in the SGED group (*p* < 0.05) (Table 2). Nevertheless, feeding SGED to rats for 33 days revealed no obvious histopathological abnormalities in all tissues obtained from these animals (data not shown).

**Table 2:** Effects of SEGD dietary intervention on body weight, fat accumulation, and organ indices in rats.

Parameters	ND group	SGED group
Total feed intake (g)	1388.95 ± 3.78	1382.80 ± 2.81
Initial body weight (g)	50.3 ± 1.59	53.9 ± 1.65
Final body weight (g)	126.2 ± 3.1	102.4 ± 3.3*
Body weight gain (g)	76.7 ± 2.79	48.4 ± 2.14*
Visceral fat (g)	0.12 ± 0.01	0.54 ± 0.04*
Visceral adiposity index	0.1 ± 0.01	0.55 ± 0.05*
Lung weight (g)	1.1 ± 0.04	0.78 ± 0.05*
Pulmonary index	0.9 ± 0.04	0.8 ± 0.06
Liver weight (g)	4.1 ± 0.13	3.5 ± 0.12*
Hepatosomatic index	3.3 ± 0.14	3.5 ± 0.12
Kidney weight (g)	0.76 ± 0.06	0.68 ± 0.06
Renal index	0.6 ± 0.05	0.63 ± 0.05
Spleen weight (g)	0.57 ± 0.02	0.59 ± 0.04
Splenic index	0.45 ± 0.02	0.55 ± 0.02*

Values are the mean ± SD (ND group, *n* = 36; SGED group, *n* = 60). * represents statistically significant differences with a *p*-value less than 0.05.

### 3.3 Effect of consuming SGED on serum lipid profile, glucose, and liver enzymes level

**Figure 2:**
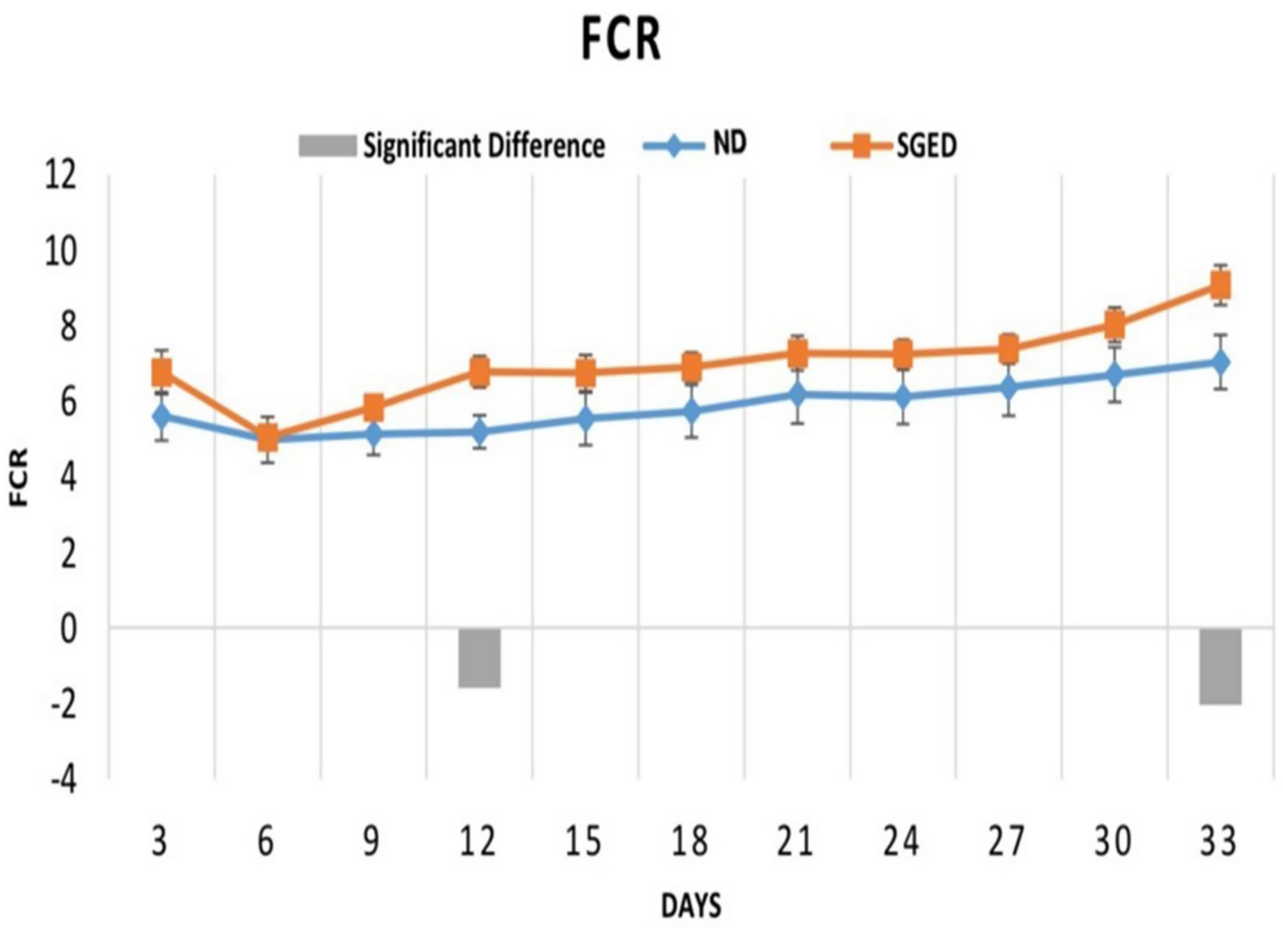
The 3-days feed conversion ratio of the consumed feed in response to different types of diets (the sugar-ghee enriched diet (60 rats) and the normal diet (36 rats)). Every three rats were placed in one cage and were provided as a group with chow. The results were presented as average FCR every 3 days for each cage. The consumed chow was calculated daily but the feed conversion ratio was calculated on average every 3 days. Each time point represents the mean of the FCR ± S.E. The significant difference (SGED-ND) with a *p*-value less than 0.05 is presented by columns in the figure.

There are several differences in the concentrations of lipids and lipoproteins in the serum. It was found that the rats that consumed a SGED showed high levels of cholesterol and triglycerides in the serum. Whereas, interestingly the levels of LDL and HDL were significantly lower compared to the group of rats that consumed the normal diet (Table 3), the LDL to HDL ratio was significantly higher in the SGED group compared to the ND group. Further analysis of lipid risk markers revealed a significant elevation in the Atherogenic Index (AI) in the SGED group compared to controls (Table 3), shifting from a negative value in controls to a positive value in SGED-fed rats. This indicates a marked alteration in lipid balance associated with SGED consumption. Additionally, the SGED-fed rats demonstrated significantly elevated levels of VLDL (Table 3), derived from the triglyceride values using standard estimation formulas. These findings further underscore the dyslipidemic profile induced by SGED, occurring in the absence of obesity and despite overall weight loss. The measurement of serum glucose level after fasting about 12 h (FBS) increased in animals that received SGED compared with animals fed normal diet (ND) (Table 3). In addition, feeding SGED to rats for 33 days showed elevated serum lipase levels (Figure 2). Rats consuming a SGED showed no significant changes in AST and ALT levels comparing to the basal diet rats (Table 3).

**Table 3:** Effects of SEGD dietary intervention on metabolic and biochemical markers in rats.

Parameters	ND group	SGED group
Cholesterol (mg/dL)	100.1 ± 4.43	127.24 ± 4.78*
Triglycerides (mg/dL)	84.867 ± 7.11	130.49 ± 6.65*
LDL(mg/dL)	35.53 ± 1.02	23.36 ± 0.59*
VLDL (mg/dL)	16.97 ± 1.42	26.10 ± 1.33*
HDL (mg/dL)	57.85 ± 4.81	33.01 ± 3.69*
LDL/HDL ratio	0.58 ± 0.04	0.70 ± 0.03*
Atherogenic index	−0.19 ± 0.06	0.23 ± 0.06
Glucose (mg/dL)	95.12 ± 6.20	58.73 ± 5.82*
Lipase (mg/dL)	78.96 ± 5.88	117.53 ± 12.78*
AST (mg/dL)	128.80 ± 16.33	139.65 ± 18.77
ALT (mg/dL)	12.10 ± 0.62	11.38 ± 1.01

The rats were fasted for 12 h before slaughter and blood was collected. Values are the mean ± SD (ND group, *n* = 36; SGED group, *n* = 60). * represents statistically significant differences with a *p*-value less than 0.05.

### 3.4 The immune response outcome after feeding SGED

**Figure 3:**
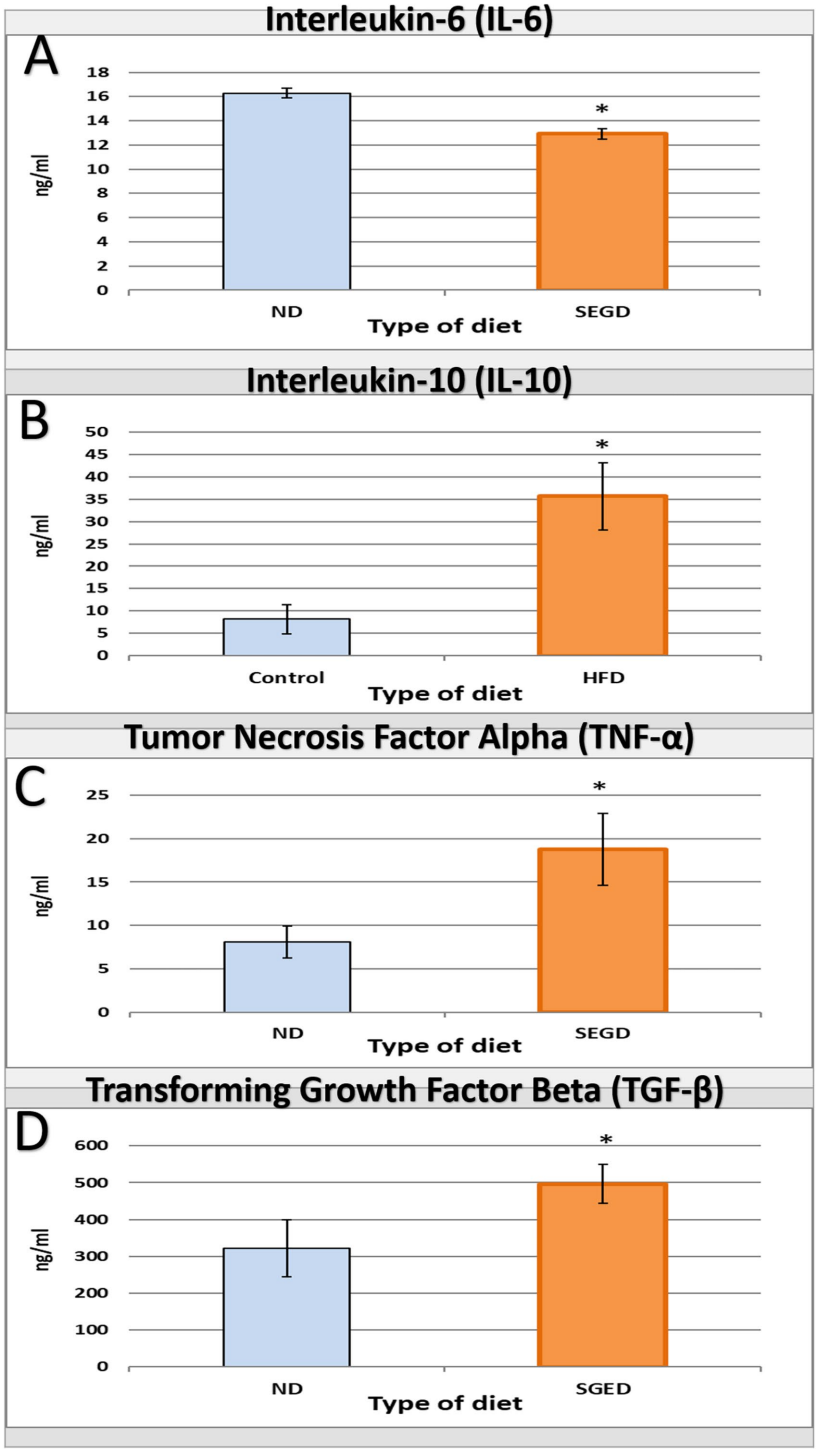
Interleukin-6 (IL-6), interleukin-10 (IL-10), tumor necrosis factor alpha (TNF-*α*), and transforming growth factor beta (TGF-*β*) levels as a result of feeding different diets for 33-days to rats. Rats were divided into two groups the SGED group (60 rats) and the ND group (36 rats). **(A–D)** A direct ELISA procedure was conducted to measure the concentration of serum **(A)** IL-6, **(B)** IL-10, **(C)** TNF-*α*, and **(D)** TGF-*β*. The results presented the average concentration of serum cytokines ± S.E. * represent statistical significant differences with a *p*-*value* less than 0.05.

The impact of SGED on immunity was represented by the measurement of cytokines released in serum by ELISA. The serum IL-6 concentration in rats received SGED showed a significant down-regulation of this cytokine concentration below the normal level present in rats’ serum received normal rat feed (Figure 3A). Other tested cytokines (TNF-*α*, IL-10, and TGF-*β*) were all up-regulated in rats fed SGED above the baseline concentration detected in control rats. Interestingly the level of IL-10 in rats’ serum fed SGED was three times more than the control level (Figure 3B), while TNF-α and TGF-*β* levels were doubled in rats fed SGED compared to rats fed normal basal diet (Figure 3C,D, respectively).

## 4 Discussion

This study introduces a novel dietary model in rats (i.e., SEGD) that simulates misapplications of ketogenic dieting and is represented as one form of pseudo-ketogenic diet (PKD) often observed in unsupervised or trend-driven human dieting behavior, which is not addressed in any previous studies. Unlike the CKD, which strictly adheres to a macronutrient distribution that induces ketosis, the SGED is high in animal fat and includes added sugar, has a ketogenic ratio = 0.21:1, which falls way below any of the known ketogenic thresholds (Table 1). Additionally, and in contrast to conventional, ketogenic, or high-fat diet models which typically rely on mixtures of plant and animal fats and minimal sugar content (14–17), the PKD pattern used fat exclusively from animal sources (ghee) and included a moderate amount of sugar. Interestingly, this combination in PKD contents did not significantly affect rats feed consumption, instead produced a higher feed conversion (FCR) than normal diet at multiple time points, indicating increased food intake without corresponding weight gain (Figure 2). This suggests a mismatch between caloric intake and efficient weight gain, potentially reflecting inefficient nutrient utilization or altered metabolic prioritization, a phenomenon observed in diets rich in saturated fats and sugar (18).

Despite inducing weight loss, the SGED led to a more than 7-fold increase in visceral fat when comparing the average visceral fat in SGED-fed rats, which was 1.22 g, compared to 0.17 g in controls, which is considered a central feature of metabolic risk. At early-stage metabolic syndrome in humans, a shift toward metabolically detrimental fat partitioning, which mimics this paradoxical accumulation of visceral fat despite reduced body weight in rats fed SGED, which is often linked to the development of insulin resistance (19, 20). Therefore, the SGED’s high sugar content and ghee’s fatty acid profile likely contributed to insulin-mediated lipogenesis in visceral fat depots, while maintaining lipolysis in other areas (21). In other words, the SGED’s combination of saturated animal fat and added sugar probably preserved lipolysis overall in the body while maintaining lipogenesis in visceral depots, which likely facilitated weight reduction alongside central fat accumulation in the visceral area. Therefore, this phenomenon mirrors the clinical phenotype of “normal weight obesity,” where individuals exhibit visceral adiposity despite a normal BMI (22).

The outcome of the current proposed PKD, along with the studies where subjects apply improper dietary practices, including individual adopting CKD, can explain the rebound weight gain for those subjects and their development of metabolic syndrome, even after they were able to maintain a low total body weight (23). Therefore, the SGED’s high sugar content and ghee’s fatty acid profile likely contributed to insulin-mediated lipogenesis in visceral fat depots, while maintaining lipolysis in other areas. This imbalance may also explain the paradoxical fat redistribution observed in this model (21).

Even though, the overall body weight remained lower in SEGD-fed rats compared to controls, the visceral adiposity index (VAI) was also significantly elevated in the SEGD rat group. This dissociation between weight and visceral fat is a known hidden metabolic threat that has been associated with increased abdominal fat in individuals after misapplied or discontinued ketogenic dieting (24, 25). The increase in VAI may also reflect a dysregulated hunger-satiety axis, as excessive visceral fat has been associated with frequent hunger, irregular eating, and altered energy metabolism (24–27), all of which were evident in the elevated feed intake observed in SGED-fed animals. Therefore, the SGED may represent a biased, palatable diet that promotes continued feeding without satiety, inducing a state similar to metabolic starvation despite caloric intake.

The current study observed that the lipid profile analysis showed significant increases in total cholesterol and triglycerides, accompanied by a shift toward a more atherogenic LDL/HDL ratio (Table 3). This is part of the development of metabolic syndrome. These changes resemble lipid imbalance commonly observed in humans consuming diets rich in saturated fats and refined sugars. Interestingly, even with high refined sugar content in SGED, rats on this diet exhibited significantly lower blood glucose levels compared to controls (Table 3). The current data point to reactive hypoglycemia, a condition often seen in the type 2 diabetes and dysregulated diets (28). This paradox may result from exaggerated insulin responses to sugar intake when combined with saturated fats, a mechanism previously described in early insulin resistance models (29). Reactive hypoglycemia is known to stimulate non-esterified fatty acid (NEFA) release and hepatic lipid build-up, processes that contribute to hypertriglyceridemia and HDL suppression (28, 30–33). These findings suggest that the SGED may induce a metabolic state characterized by dyslipidemia and impaired glucose homeostasis, despite its high sugar content.

In support of these findings, the calculated atherogenic index (AI) and estimated VLDL levels further confirmed the pro-atherogenic lipid environment induced by SGED. Despite lower total body weight, the SGED group demonstrated a higher AI and VLDL concentration compared to controls, both indicative of increased cardiovascular risk and dysregulated lipid metabolism. Notably, the AP, which was negative (−0.19) in the ND group, shifted to a highly positive value in SGED-fed rats, indicating a transition from a metabolically healthy state to one of elevated risk which outlined to have AI above 0.24 (34). Very low AP values (< 0.1) are typically observed in metabolically healthy states, and slight negative values are physiologically plausible when TG/HDL < 1. AP classifications, including ‘extremely low’ (< −0.3) and ‘low’ (−0.3 to 0.1), are described in the literature, even though an overall negative mean is rarely documented (34). A shift from such low values into the positive range after receiving SEGD (0.23) generally emphasizes the medium to high risk of cardiovascular risk. In general, markers as AI and VLDL are frequently used as predictors of metabolic syndrome and can be strongly linked to visceral fat accumulation and insulin resistance in both experimental and clinical settings (18, 35). This reinforces the notion that SGED promotes metabolically harmful lipid shifts even in the absence of overt obesity, highlighting the deceptive metabolic profile of pseudo-ketogenic dietary patterns.

Histological and biochemical markers provided further insights. Liver and lung weights were significantly lower in SGED-fed rats compared to the control group; however, liver histology and hepatic enzyme levels (ALT, AST) remained within normal ranges, suggesting no clear hepatotoxicity (Table 3). These findings contrast with prior studies showing that high-fat diets combining plant and animal fats often lead to fatty liver, organ hypertrophy, or damage in the kidney, heart, and spleen (36–43). This dissociation indicates that metabolic and immune disruptions precede visible tissue pathology, underscoring the model’s relevance in simulating early, reversible stages of metabolic disease. Such preclinical manifestations are analogous to “silent” phases of metabolic syndrome and non-alcoholic fatty liver disease (NAFLD) in humans (44, 45).

The immunological profile observed in SGED-fed rats showed an upregulated TNF-*α*, IL-10, and TGF-*β* and lower IL-6 production compared with the control group, which reflects a non-classical, mixed immune response. TNF-*α* is connected to adipose inflammation and metabolic syndrome (46), while IL-10 and TGF-*β* are regenerative cytokines often upregulated in response to fat-induced immune activation and pancreatic stress (47–51). The reduction in IL-6, a pro-inflammatory cytokine, that is typically elevated in obesity models, can be related to reduced carbohydrate intake and lower glycemic flux, as IL-6 secretion is closely linked to glucose metabolism and hyperglycemia (52–56). This cytokine expression pattern in the SEGD fed rats suggests a state of early metabolic stress rather than classical obesity-driven inflammation. Supporting this interpretation, the splenic index was significantly elevated in SGED-fed rats (Table 2), despite the absence of clear splenic pathology. Splenomegaly has been documented in high-fat and high-sugar diet models and is frequently associated with increased levels of circulating inflammatory mediators, particularly TNF-*α* (42, 57–59). The rise in TNF-*α* and splenic mass implies low-grade immune activation which is consistent with chronic metabolic stress. Moreover, the simultaneous increase in IL-10 and TGF-*β*, and reduction in IL-6, point to a compensatory anti-inflammatory feedback likely aimed at restoring immune imbalance.

It is important to mention that serum lipase levels were significantly elevated in SGED-fed rats (Table 3), indicating increased pancreatic enzymatic activity. Although histological evaluation revealed no signs of overt pancreatitis, the biochemical and cytokine findings raise the possibility of ongoing or subclinical pancreatic stress. TNF-*α* has been linked to early pancreatic inflammation (27, 60), and its co-existence with elevated lipase suggests that the SGED may stimulate exocrine pancreatic activity in a manner consistent with early, undetected pancreatic injury. Previous research has shown that diets high in saturated fats can increase pancreatic enzyme release and inflammatory signaling without causing histopathological lesions (61). Thus, these findings suggest that the SGED triggers a state of immune metabolic dysregulation, with features of early immune activation and potential pancreatic stress, even in the absence of obesity or organ damage. This shows the SGED’s can be proposed as a behaviorally relevant model for studying the hidden consequences of pseudo-ketogenic dietary practices.

In summary, SGED-fed rats consumed a palatable animal fat–sugar combination mimicking flawed ketogenic practice, leading to paradoxical weight loss with increased visceral adiposity, dyslipidemia, cytokine imbalance, and early immune activation. To our knowledge, this is the first animal model that explicitly simulates pseudo-ketogenic dietary behavior, distinguishing it from traditional high-fat or high-sugar models. It offers a behaviorally relevant and mechanistically distinct framework for studying the consequences of flawed ketogenic mimicry, a growing concern in unsupervised dieting and social media–driven trends.

## 5 Conclusion

This study shows that the sugar-ghee-enriched diet (SGED), which is designed to mimic the behavior of individuals adopting pseudo-ketogenic dieting, causes significant metabolic and immune alterations in rats, even though it helps in losing weight. SGED does not reach the ketogenic macronutrient threshold like real ketogenic diets do, but it does imitate common mistakes people make when they combine high-fat foods with added refined sugar. Rats that were given SGED gained more visceral fat, had higher triglycerides and cholesterol levels, and had lower blood sugar levels. This shows that losing weight does not always mean better metabolic health. At the same time, the cytokine profile revealed a complicated immunological response, with both pro- and anti-inflammatory mediators being upregulated with an increased splenic index, suggesting subclinical immune activation. These findings occurred without clear liver or kidney histopathological changes, which represents the model’s ability to capture early stage metabolic dysregulation. To our knowledge, this is the first animal model developed specifically to simulate pseudo-ketogenic dietary practices. These findings raise concern about the health implications of unsupervised or poorly structured ketogenic diets and underscore the need for greater awareness regarding their metabolic consequences. Further research is warranted to explore long-term outcomes and the mechanistic pathways underlying these effects, including comparative studies with classical ketogenic diets to better delineate the distinct metabolic and immunological consequences of pseudo-ketogenic patterns.

## Data Availability

The raw data supporting the conclusions of this article will be made available by the authors, without undue reservation.

## Footnotes

^1^ http://www.openepi.com/Menu/OE_Menu.htm
